# Reconceptualizing Somatic Dysfunction in the Light of a Neuroaesthetic Enactive Paradigm

**DOI:** 10.3390/healthcare11040479

**Published:** 2023-02-07

**Authors:** Giacomo Consorti, Carmine Castagna, Marco Tramontano, Mauro Longobardi, Paolo Castagna, Daniele Di Lernia, Christian Lunghi

**Affiliations:** 1 Education Department of Osteopathy, Istituto Superiore di Osteopatia, 20126 Milan, Italy; 2 Fondazione Santa Lucia Istituto di Ricovero e Cura a Carattere Scientifico, 00179 Rome, Italy; 3 Centre Pour l’Etude, la Recherche et la Diffusion Osteopathiques, 00199 Rome, Italy; 4 Italian Register of Osteopaths, 20144 Milan, Italy; 5 Human Technology Laboratory, Università Cattolica del Sacro Cuore, Largo Gemelli, 1, 20100 Milan, Italy; 6 Osteopatia Lunghi-Baroni Private Practice, 00146 Rome, Italy

**Keywords:** somatic dysfunction, neuroaesthetic, Bouba/Kiki-effect, touch, shared decision making

## Abstract

**Background:** Palpatory findings are considered a central element of osteopathic practice, especially when associated with a patient’s altered regulative functions than with named somatic dysfunctions. Although osteopathic theories for somatic dysfunction could be plausible, the clinical applicability of the concept is debated, especially because it is largely related to simple cause–effect models of osteopathic care. In contrast to a linear kind of diagnosis of a “tissue as a producer of symptoms”, this perspective article aims to provide a conceptual and operational framework in which the somatic dysfunction evaluation process is seen as a neuroaesthetic (en)active encounter between osteopath and patient. **Subsections relevant to the subject:** To summarize all concepts of the hypothesis, the enactive neuroaesthetics principles are proposed as a critical foundation for the osteopathic assessment and treatment of the person, specifically addressing a new paradigm for somatic dysfunction. **Conclusions, and future directions:** The present perspective article represents a proposition to blend technical rationality informed by neurocognitive and social sciences, and professional artistry clinical experience informed by traditional tenets, to overcome the controversy around somatic dysfunction, rather than dismissing the concept.

## 1. Introduction

### 1.1. Somatic Dysfunction: History, Evolution, Definition and Research

**Figure 1:**
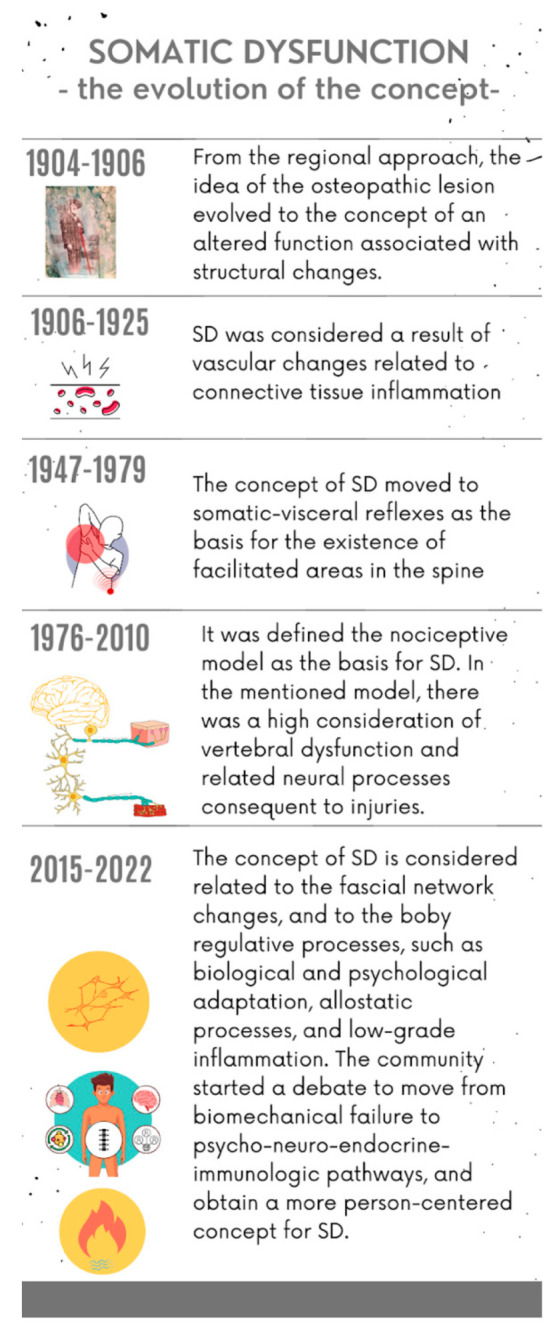
Somatic dysfunction: the evolution of the concept.

Somatic dysfunction (SD) is a key element of osteopathic practice [1]. SD was originally mentioned as osteopathic lesion (OL) by A.T. Still, the first osteopath [1]. The term OL was dismissed and changed to SD in 1968 by a working group of the Educational Council on Osteopathic Principles (ECOP), led by Ira Rumney and Norm Larson [1]. The Hospital Assistance Committee of the Academy of Applied Osteopathy, chaired by Ira Rumney, DO, developed definitions for osteopathic diagnosis and treatment, including SD, for inclusion in the Hospital International Classification of Disease (ICD) [1]. To date, SD is still listed in the present version of the ICD [2]. From being considered a milestone of early osteopathic regional anatomical approaches to the person, it has come to be viewed as body regulative function activity impairments related to the body framework, a region, or a generalized body schema that involves the whole organism, resulting in the “environmental lesion”, or “total lesion”, and “greater osteopathic lesion complex” [1]. During the evolution of the concept, SD was considered to have developed as a result of vascular changes related to connective tissue inflammation; or as a result of somatic-visceral reflexes as the basis for the existence of facilitated areas in the spine, representing the starting point of the nociceptive model [1]. A following mechanistic spine-centric model of health and disease impressed a huge portion of the community of practice [3], resulting in the adoption of a misinterpretation of the original concept of somatic dysfunction. Finally, osteopaths recovered the concept of the connective tissue framework, named fascial network, as a substrate related to biological and psychological adaptation, allostatic processes [3], and low-grade inflammation [4]. Consequently, the osteopathic community started a debate to move from biomechanical failure to psycho-neuro-endocrine-immunologic pathways and obtain a more person-centred concept for SD [5]. The Glossary of Osteopathic Terminology defines SD as an “*Impaired or altered function of related components of the body framework system: skeletal, arthrodial and myofascial structures, and their related vascular, lymphatic and neural elements. It is characterized by positional asymmetry, restricted range of motion, tissue texture abnormalities, and/or tenderness*” [6]. The latter listed parameters fall under the acronym of TART and have been referred to as the “diagnostic criteria” for SD. The evolution of the somatic dysfunction concept is summarized in Figure 1. Nowadays, on the one hand, a part of the community of practice is asking to dismiss the concept [7]. On the other hand, another part of the community proposes a reconceptualization of SD, considering the clinical entity as a pivot point that could favour the establishment of meaningful connections with the patient by using the body and the associated regulative biological and psychological functions [8] (see Supplementary Materials. Table S1: Somatic dysfunction, historical overview).

### 1.2. Rationale and Objective

The evolution of the SD concept culminated with the need to overcome polarized exclusive models of practice, such as examples solely focused on biomechanical or psychosocial functions to drive management strategies and related explanations [8] and a focus on obtaining a shared, culturally sensitive framework that can respect the different patients’ health sociocultural assumptions with more person-centred osteopathic care [9]. A first attempt to synthesize the combination of manual assessment and related alteration of function was proposed by Castagna and colleagues [8]. However, it included not only the interpretation of SD but also its contextualization with the osteopathic structure/function models. Such an all-embracing process could have aroused the interest of treaders in the relationship between the palpatory findings and the impaired functions underlying the osteopathic clinical decision-making, rather than on the patient’s responsiveness and perceptions.

Recently, through the enactive model, the patient has been reintroduced in the clinical process [10,11,12,13] and the operator touch has been included as an integrative way of non-verbal communication. The proposed models focused particularly on the therapeutic value of the practitioner-patient relationship mediated by touch, but the diagnostic value of the therapeutic alliance is scarcely mentioned. 

Going from the scientific reports to the daily practice field, results from a focus group published in 2022 highlighted different beliefs and the use of palpatory findings in osteopathic clinical practice. On the one hand, some experts consider SD as a clinical entity to assess by an operator-dependent ability; on the other hand, other panelists claim the need to evaluate the SD through a shared process in the operator–patient relationship. The authors suggested future international consensus conferences to achieve a shared framework [14]. A recently published qualitative study explored Italian experienced osteopaths’ attitudes concerning SD and its role in osteopathic practice [15]. The point of view of participants was that SD is a safe touch-based communication tool between two complex adaptive health systems, the osteopath and the patient. According to Consorti and colleagues [16], participants (who were patients) considered the osteopaths as facilitators who accompanied patients in the discovery of unexpected bodily connections, including psychological and emotional ones. Moreover, they stated that during the osteopathic clinical encounter the osteopath “*starts a communication with the patient’s body*”.

The panel of experienced osteopaths, participants in the Arcuri and colleagues’ qualitative study [15], describe SD as the result of a touch-based participatory interaction process between the osteopath and patient, which is also shared through respectful verbal conversation. According to the attitudes of the respondents, SD should be seen as an emergent pattern of body framework and systems interdependence that informs the sense-making of the different complexity domains of the daily clinical scenario. Furthermore, the role of SD in osteopathic diagnostic-clinical reasoning is mainly to address the touch input quality in the region of the entire body to improve biological and psychological self-regulation, focusing on patient agency, body awareness, and adaptive capacity. Participants declared that they “*only take SD into account if clinically reflects the expression of an altered functioning of the patient*”. The attitudes of the participants concerning SD seem to be informed by the recent proposals on the role and application of palpatory findings in the osteopathic field [3,5,8,17,18]. At the basis of the process, there seems to be the operator’s perception of an observed and felt disharmony in one or more body regions [19]. The operator tries to confirm or refute their intuition through the pleasant or unpleasant sensations of the patient while exercising different types of touch and requiring the execution of familiar movements [19]. When there is an emergent perceptual concordance in a region, the osteopaths look into the potential involvement of the body systems [5,8,17], the related homeostatic–allostatic processes [3], and psychosocial–existential aspects [20]. Results from the thematic analysis showed that in the current time there are different mindlines concerning the conceptual basis of SD [15]: there are members of the osteopathic community of practice that do not consider SD as the outdated clinical entity that is criticized in the current debate [7]. For example, Italian osteopaths do not consider SD as a way to detect and resolve objective structural signs at the origin of the patient’s symptoms, nor a cornerstone of a simple cause–effect model of osteopathic care [15]. A survey published in 2018 showed that patients with musculoskeletal disorders who had personalized osteopathic care, also focused on the concept of somatic dysfunction in a hospital environment in Italy, reported a high degree of overall satisfaction [21].

To date, there is no available framework that aims to integrate the systematic evaluation of SD in all the above-mentioned aspects: (a) interpretation of palpatory findings in the light of patient responsiveness; (b) co-definition of their meaning in the patient’s lived experience; and (c) evaluation of their impact on one or more functions.

Therefore, we propose an integrative clinical mindline aimed at guiding osteopaths in the osteopathic evaluation of SD and the related treatment approach. The proposal will be followed by clinical examples to help the readers to ground the theoretical concept expressed.

## 2. Proposal

The process of assessing SD is a sensory perception (in particular by vision and palpation) that evokes an “unpleasant” sensation. Patients are perceptually involved in this process since they are required to validate or refute osteopaths’ perceptions. The neurologic assumption of the “pleasant/unpleasant” sensation will be considered according to the neuroaesthetics concept [22]. 

This manuscript aims to propose a conceptual and operational framework in which the SD assessment process is considered as a neuroaesthetic enactive encounter between osteopath and patient, rather than a cause-effect type of diagnosis of a tissue causing symptoms.

The proposed framework allows osteopaths and patients to enhance a collaborative development of terms anchored in multi-sensory correspondences which guide a shared sense-making process. 

### 2.1. Neuroaesthetics for Health

The word “*esthetic*”, derived from the Greek word “*aesthesis*”, refers to sensory perception in general, and encompasses more than simply visual perception. It also refers to the physical imprint that the perceived drives on the body. Tactile, visual, olfactory, and hearing perceptions make up a whole in the concept’s original sense and contribute to the aesthetic experience, together with discernment. An aesthetic encounter is something perceived by the entire body. That results in something positive just by the fact that our sensory, motor, and cognitive systems are engaged with the environment, the people around us, and the context we are in. 

By evaluating neuroaesthetics in an evolutionary key, we can explain why the brain would not generate aesthetic reactions if a useful purpose for survival could not be achieved. Neural processes are strongly selected if they maximize survival. Indeed, from the very beginning, babies between two and three months of age show aesthetic reactions to faces [23,24] and three-year-olds have preferences for friendship based on the perceived beauty of the face [25]. Since these preferences could not have been learned so early, nor the concept of beauty itself, this ontogenetic evidence suggests a biological and adaptive function fundamentally useful for aesthetics, which goes beyond simple pleasure/displeasure and can embody an adaptive and evolutionary value [26].

Understanding the neurological mechanisms underpinnings aesthetic sensations is the focus of neuroaesthetics [22]. Perception, emotion, semantics, attention, and decision-making are some of the more well-known cognitive neuroscience topics that are influenced by and drawn from neuroaesthetics [22]. Neuroaesthetics was founded by Ishizu and Zeki, who discovered the link between the brain and pleasant and unpleasant stimuli, beauty and ugliness [27]. They found that participants’ brains showed higher activity when they listened to music, looked at an image or touched something that they had already judged to be pleasant, or beautiful. The medial orbitofrontal cortex is known as the pleasure and reward centre of the human brain [27]. Ishizu and Zeki discovered by looking at magnetic resonance imaging scans of their patients’ brains that people who found something attractive experienced increased blood flow in the medial orbitofrontal cortex [27]. The authors think that almost everyone reacts to beauty in this way. It effectively conveys to us that the desire for beauty leads to a universal state of “feeling well”, and that there are sometimes universal routes to achieving it [27]. This “feeling well” sensation seems to be mediated by dopamine, which “rewards” the brain’s pleasure centre. According to Ishizu and Zeki, beauty experienced through the senses (e.g., visual, haptic, hearing perceptions) doesn’t route to different brain areas. Instead, they all “reward” the same area [27]. The level of activity in the medial orbitofrontal cortex substantially correlates with how attractive you find something [27]. Neuroaesthetics concerns a wide spectrum of aesthetic experiences, resulting from interactions of individuals, sensory stimuli, and context. Does it concern the osteopath-patient relationship? 

### 2.2. Neuroaesthetics for Osteopathy: A Journey from Unpleasant-Pleasant Perceptions to SD (Phases 1 and 2)

**Figure 2:**
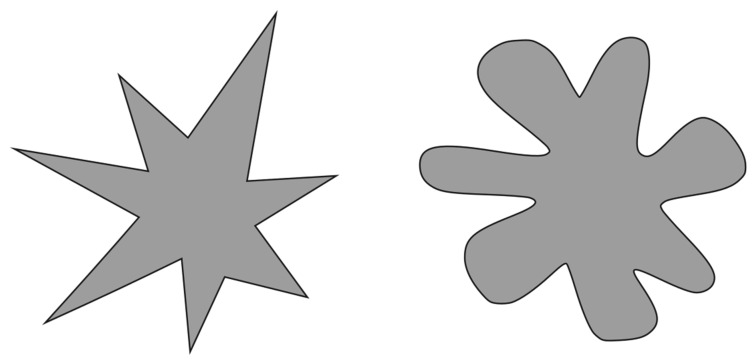
Kiki and Bouba.

Aesthetic experience is enacted and skillful [28]. It is founded on the recognition of others’ experiences as different from one’s own. We all have bodies, which is the only reason physical objects have personalities. We would never be able to judge the aesthetic worth of the physical world if we were exclusively visual beings. Furthermore, because we are humans with bodies that educate us about gravity, contraction, strength, and other physical phenomena, we obtain the experience necessary to be able to relate to the conditions of other forms [28]. Aesthetic experience emerges through participatory sense-making and revolves around movement as a means of creating meaning [28]. It is common knowledge that stimuli from the external world can be evaluated on an abstract spectrum by Bouba/Kiki-effect (BKe), with “Kiki”, spiky, disharmonious, incongruent and unpleasant on one hand and “Bouba”, rounded, harmonious, congruent, and pleasant on the other (Figure 2). Experiments over the last 90 years have shown that 95% of the world’s population are in concordance with each other concerning associating a rounded, symmetrical, pleasant visual shape with the spoken word “Bouba”, and an angular, asymmetrical, unpleasant visual shape with the spoken word “Kiki”, known as the Bouba/Kiki-effect. The BKe occurs in adults, infants, toddlers, and children, irrespective of familiarity with either the shape or word [29]. It appears that “Boubaness” or “Kikiness” is a universal concept. Studies have shown that cross-modal associations between and within the senses of sight, smell, hearing, and taste, can be demonstrated by using Bouba-Kiki as a cognitive “convergence point”. 

The fundamental clue emerging from the BKe demonstrates that there is a pre-existing unarbitrary translation of the visual aspect of an object (in the fusiform gyrus) into an acoustic representation (in the auditory cortex), tactile (somatosensory cortex). By acting together, the tactile, visual, auditory and—probably—olfactory cross-activations have a synergistic trigger effect: an avalanche that culminates in the emergence of a primitive language, probably already used by our hominid ancestors to represent pleasant or unpleasant, friendly, or hostile circumstances. Research on multisensory integration showed non-arbitrary associations of cross-modal correspondences involving the tactile modality: participants touching different surfaces associated smoother textures with Bouba, whereas rougher textures were more strongly associated with Kiki [30]. Additionally, when using haptic touch, with or without including visual and mental imagery, there is an instant BKe [31]. 

From a clinical practice standpoint, we propose to use the neuroaesthetics paradigm to guide the palpation process. The aim of osteopathic palpatory evaluation, in this case, is to find anatomical areas that “feel like Kiki”. There is no fixed way to define what the tissue should feel like; bogginess, thickness, ropiness, stringiness, firmness, and any changes in temperature and moisture are all possible palpatory findings. However, from a neuroaesthetics point of view, the focus is not the palpatory feeling itself, but rather the sensation evoked while the osteopath palpates in terms of “I like it vs. I do not like it”. A fundamental aspect of “finding Kiki” is that it is a process which involves the patient. It is fundamental that “Kiki” is recognized as Kiki by the patient and not only by the osteopath. It might happen that what feels like “Kiki” for the osteopath feels like a good kind of “Kiki” for the patient. This situation is often described by patients as “*a good pain*” or “*it hurts but I am feeling that it does the job!*” [32]. That opens up a window of possibilities for the treatment where the Kiki spot is possibly *Boubazable*. According to Kim and colleagues [32], attention-based strategies, such as mindfulness-based interventions, might lessen pain management. This leads to seemingly incongruent findings about attention and pain. The main somatosensory cortex and goal-directed attention areas in the prefrontal and parietal cortex mediate the analgesic benefits of attending to painful stimuli. These results provide evidence that pain itself can be employed as a component of pain management by pointing to top-down regulation as the main mechanism of the analgesic effects of paying attention to painful stimuli [32]. The fact that patients have an active role in attributing significance to a palpatory finding helps in foraging their body awareness (which is one of the indicators that patients monitor to determine the effectiveness of an osteopathic treatment according to a recent article [16]). Moreover, some authors [33] have hypothesized a relationship between body awareness and autonomic homeostatic processing. Indeed, the main subcortical neural substrates for these processes are limbic-related systems.

In this first phase, the osteopath, with the patient’s help, generates a hypothesis regarding the presence of an SD. Indeed, the palpation alone does not address the “impaired function” but only the possibly associated areas; therefore, it is not possible, at this moment, to diagnose an SD. To confirm or disconfirm the hypothesis, there must be a further phase. Due to the active role of the patient in the entire proposed process, we would rather refer to the patient as an “agent”. However, for clarity purposes, to avoid any possible misinterpretation, we will address the patient as a patient in the present perspective article.

### 2.3. Enactive Neuroaesthetics Concept during the Osteopathic Evaluation of SD (Phase 3)

The present phase consists of a battery of tests aimed to correlate the palpatory findings (which emerged by the patient in the previous phase) to an insufficient patient agency (i.e., the ability to do usual actions in daily life) and/or to an altered or impaired function (both biological and psychological).

First, we must answer the question: “what impaired or altered function do we observe?”

It goes without saying that it depends on the clinical presentation of the patient:

If the patient has a clinical presentation which is elicitable through provocation tests (e.g., a specific movement that elicits their symptom) it is possible to use that. It takes the name of familiar symptoms (reported by the patient as related to the chief complaint) and comparable signs (physical examination findings related to the chief complaint). A tender point which evokes the exact kind of pain the patient comes for might be considered a neurological altered function (familiar symptom) and therefore can be used.

If a semeiotic test (e.g., straight leg raising test, eliciting Lasegue’s sign [34]) is positive on a patient it is possible to use that.

If the patient is asymptomatic, it is possible to use functional tests (e.g., one leg standing [35]; joint position sense error [36], and other functional physical examination tests useful in the osteopathic practice [8]).

In the cases of osteopathic encounters with fragile people, for example, older adults with chronic pain, the positivity of SFCT implementing familiar symptoms, comparable signs, and functional tests will guide the osteopath in considering the multidimensional conceptual model of frailty [37]. It has emerged that frailty is a loss of harmonic interaction between multiple dimensions, such as genetic, biological, functional, cognitive, psychological, and socio-economic domains, that lead to homeostatic instability. Therefore, implementing a comprehensive geriatric assessment, such as the multidimensional prognostic index (MPI), could help capture the multidimensional aspects of frailty conditions [37]. Consequently, osteopaths might consider negotiating with the patients and their families on the proposal of homecare services [38]. Combining the most important life domains (i.e., health, family life, and friendships) and gradually exposing the patient to daily activities in the customary context could enhance the potential biopsychological benefits of osteopathic care.

Secondly, we need to answer the question: “how do we correlate those functions with a palpatory finding?”

We propose to use structure–function correlation test (SFCT) (previously known as Inhibitory test) [39]. In these tests, the osteopath applies a touch-based stimulus to an area that was retrieved in phases 1 and 2, and while the stimulus is maintained, the function that was previously assessed is retested. The test is positive if the functional test becomes negative or if it is possible to observe positive changes.

The next question we need to answer is: “what kind of touch-based stimuli should the osteopath apply?”.

There are different types of stimuli that it is possible to apply to different regions of the body. There is no fixed way to assess *a priori* what kind of touch is the most indicated. The stimulus can be a pressure, a strain (in different directions), a light slow stroke (interoceptive touch possibly correlated to the activation of C-tactile fibres) or an active muscle activation request.

Different kinds of touch lead to different types of neurophysiological mechanisms underlying SFCT. Three separate mechanisms are presumably activated when this type of test is applied [39].

(1) The proprioceptive touch: mechanical stimulus provided by touch can activate tactile and proprioceptive mechanoreceptors that have low activation thresholds [19]. The mechanoreceptors activate thick-type myelinated fibres of type I and II, or A and Aβ, with high conduction speeds, and inform the central nervous system. The posterior horn of the spinal cord receives information from large-calibre neurons more quickly and powerfully, altering the synaptic connections stimulated by the nociceptive input and temporarily altering the state of neural sensitization [19]. Dynamic-type touch, also associated with movements, provides auxiliary proprioceptive feedback for guiding actions. Proprioception, as well as exteroception, are related to the discriminative type of touch [40]. Discriminative touch supports the perception of pressure, vibration, slide, and texture, all of which are essential for haptically revealing information about handled items and for conducting exploratory activities. The main function of such systems is to recognize, classify, and distinguish between environmental stimuli in order to quickly determine which action to do next [40]. Discriminatory touch is possible on glabrous skin, such as the surface of the hand, in which there is a high density of specialised mechanoreceptors able to encode the spatial and temporal properties of surfaces and handled objects [40]. Hairless skin has different mechanoreceptors with low conduction thresholds, different shapes, and sensory properties [40]. The mechanoreceptors are connected to high-conduction speed fibres. Merkel cells record static touch; Ruffini corpuscles decode skin distension; Meissner mechanoreceptors are sensitive to movement; Pacini corpuscles are susceptible to vibrations to high frequency, and acceleration; Muscle spindles record compressions; and Golgi receptors decode slow elongation [19]. When a mechanical stimulus acts on the receptors generates an informational pathway that through the fibres reaches the spinal cord and the postcentral gyrus, where Penfield’s sensory homunculus resides [41].
(2) The interoceptive touch: the evaluator’s touch (if it has the characteristics needed to activate c-tactile fibres) in a neurologically active area induces an inhibitory reflex because this stimulus may change responses evoked by free nerve endings from a constant interpretation of “painful” harmful sensation to an interpretation of “pleasant” non-harmful sensation [19]. The activation of type C unmyelinated fibres can promote inhibition of the activity of the amygdala as well as the activation of cortical regions, such as the left insular, anterior cingulate, and left prefrontal cortex, favouring a modulation of interoceptive information and enabling adaptations of the autonomic nervous system towards a different interpretation of pain [19]. The interoceptive touch (often called affective touch) relies on specific stimulation of the peripheral C-Tactile (CT) afferent system. This system encompasses peculiar fibres that differentiate in tactile afferent receptors forming a secondary touch system [42,43,44] that is interoceptive rather than purely somatosensory [45]. Animal models indicate that CT stimulation elicits a neuro-modulatory inhibitory effect in the dorsal horn, with a concomitant release of protein TAFA4 that has analgesic and anti-inflammatory effects [46,47]. Other studies demonstrated that CT stimulation can reduce acute pain in humans [48,49,50], mediate the µ-opioids system response [51] and oxytocin release [52], which are important chronic pain therapy targets with direct effects upon pain intensity, anxiety, and depressive symptoms [53,54]. Moreover, it has been demonstrated that CT stimulation can enhance parasympathetic autonomic activity (with a possible concomitant reduction of sympathetic pain-related response) [55,56]. Lastly, interoceptive touch has been successfully applied to reduce chronic pain in clinical contexts [57]. Several studies explored the role of C-Tactile stimulation, reporting sometimes conflicting results [58]. These conflicting results can be possibly explained by the unique nature of the C-Tactile afferents, as these receptors respond uniquely to low-force, low-velocity stimuli being unresponsive to mechanical vibration, high velocities or indentation forces, which are often difficult to reproduce. A recent review by Ackerley [59] described in detail the specificity of these receptors and their unique behaviour, redefining the current literature on interoceptive touch. In her seminal work, CT receptors are specifically sensitive to slow (around 3 cm/s) dynamic touch of less than 5 mN (500 mg) and they are not “*necessarily tactile pleasantness receptors as such, but they play a clear modulatory and reinforcing role of gentle, comfortable touch interactions*” [59]. In this context, the evaluator’s touch in a neurologically active area (if applied matching the characteristics needed to activate C-Tactile fibres) could potentially activate the C-Tactile system promoting a cascade of positive effects, enabling adaptations of the autonomic, cardiovascular, endocrine, and cortical systems, therefore, modulating the perception of pain.
(3) Local and global changes in the fascial tissue: the mechanical stimulus provided by the manual contact in the neurologically active area, perceived as unpleasant in the osteopath-patient dyad, can momentarily modify the mechanical stresses of this continuous system that propagates and connects the entire human body, promoting temporary changes in the viscosity of the connective tissue [60], the fluid dynamics [61,62] (i.e., vasodilatation) and also the trigger threshold of mechanosensitive receptors present in the extracellular matrix, enabling the reduction of local sensitization [63], as well as changes that propagate throughout the fascial continuum [64,65,66,67,68].

In addition, we could speculate on recently proposed conceptual models based on active inference, the free energy principle, and enactivism [12]. We can consider the enactive-ecological Framework [12] as underlying the patient-osteopath verbal, non-verbal and proximity-touch-based interactions in general, including during the administration of SFCT. Cerritelli and Esteves [12] proposed an integrative hypothesis by presenting osteopathic care as an (en)active inference. Persistent body symptoms are typically associated with a person’s emotional and cognitive scenario and the inability to selectively ignore, pay attention to, or attenuate different sources of sensory evidence. The incapacity to re-evaluate the generally “irrelevant” bodily signals could depend on the structured predictive models not prone to change (redundant interpretation). This would result in a sustained interpretative output over time (i.e., pain) [11]. In the described clinical context, the modulation of the symptomatic state, selective sensory attention (cognition), and the possibility of promoting the adaptation and restoration of new body narratives and predictive models could be obtained through a good therapeutic alliance and musculoskeletal approaches (e.g., osteopathic care) [11,12]. Integrating hands-on, hands-off approaches and educational strategies could be appropriate, effective, and achievable care strategies, in the light of concepts such as (en)active inference, the ecological niche, and the operator–patient dyad [11,12]. The conceptual hypothesis clarifies some aspects of the osteopathic discipline, proposing a conceptual umbrella to be shared between different health professionals. The enactive model can be used to support any other healthcare professionals that contemplate models of musculoskeletal care based on informative, educational, and psychological care activities of patients (e.g., physiotherapy) [10,69]. 

According to recent proposals, osteopathic clinical reasoning is considered as informed by a multidimensional assessment to define person-centred treatment models. Emergent elements from patients’ verbal and body narratives, standardized patient-reported outcomes measures, patient-reported experience measures, functional objective examination, and palpatory findings are implemented [65]. It results in the selection of the most appropriate passive, manipulative and active approaches to promote personalized and evidence-informed treatment [32], and therapeutic patient education [70] to support patients in their different stages of life, e.g., musculoskeletal health and integrated care for older people [71]. In the light of enactivism, person-centred osteopathic care aims to enhance the patient’s agency by restoring the interdependence of psychological and biological functions [5].

The enactive-ecological framework represents the incipit for a re-evaluation process of osteopathic aims, working practices and education [10,11,12,13,22,72]. Shaw and colleagues [73] suggested that future research must assess how to introduce concepts of active inference and enactivism, participatory sense-making, and mindful self and body awareness in the osteopathic practice and education [20]. To reframe the concept of SD, we proposed a neuroaesthetic enactive paradigm to stand back from old biomechanical theories, and highlight the participative and complex nature of osteopathic palpatory findings.

Cerritelli and Esteves proposed osteopathic care as an interactive ritual that enables feelings to be reinterpreted, awareness redirected, and distracting stimuli attenuated and ignored [12]. The authors illustrated the process of perceptual inference referring to clinical scenarios in which patients may refer to sensations of ‘good pain’. Following the weighted predictions will indicate that the source of the noxious stimulation comes from the osteopathic approach. Consequently, there will occur an update of the beliefs concerning tolerance of nociceptive stimuli deriving from applying an osteopathic technique [12]. Accordingly, we propose that a participatory sense-making process begins in the osteopathic assessment procedure: in the meanwhile, the osteopath is in manual contact with an emergent perceptual concordance region, the hypothesized SD, and the patient starts having positive surprise and a high precision-weighted prediction. The osteopathic touch on the person, thanks to SD, will violate existing predictive models (for example, feeling less pain and less fear of movement in lumbar flexion after the osteopath has exercised a particular type of touch—not another—in a remote area—not another).

In our perspective, and in agreement with the (en)active inference osteopathic care model [20] informed by the osteopathic-touch neuroaesthetic paradigm [19], the (en)active-neuroaesthetic process starts in the osteopathic evaluation process. The described participative assessment of SD is based on a generative and body narrative model, shared by agents who exchange sensory signals. It is a process of dyadic participatory sense-making, informed by selective attention and the attenuation of sensory information. Proprioceptive, exteroceptive, and interoceptive awareness enables agents to anticipate the sensory input of the other. On the contrary, the attenuation of the relevant interoceptive and exteroceptive inputs allows for articulating the body narratives by realizing proprioceptive predictions (e.g., movement). The SD is not observable and detectable by the osteopaths by their ability but must be inferred: an emergent pattern from the patient will be needed (i.e., results from SFCT) [73]. All professionals in charge of individual musculoskeletal care, including osteopaths, achieve this through communication, touch, movement, and exercise treatment. The osteopath–patient dyadic relationship beginning in the palpatory evaluation generates an ecological niche beyond the body itself and produces an alignment and a bio-behavioural synchronization. In the case of the osteopath-patient agreement on pleasant perception consequent to touching an area of interest for both the agents, a positive surprise is evoked in the priors of the patient, consequently, re-designing predictive models more congruent with the ecological-social context in which the patient lives [12].

### 2.4. Selection of the Osteopathic Approaches Based on Patient Responsiveness to the SFCT (Phase 4)

The SFCT is used to assess the correlation between a palpatory finding and an altered function affecting a patient’s agency, therefore, to diagnose SDs [39] to administer a personalized treatment. The mentioned tests follow the principles of osteopathic inhibition approaches. These are functional tests, in which the evaluator generates manual stimuli in the areas of SD and evaluates the possible changes provided by this stimulus [39].

This test class is used as a driver within a shared decision-making process and allows the operator to confirm or refute his hypotheses based on the patient’s emerging responses [19]. To verify the hypothesis of the presence of SD for the patient (and disentangle it from a purely biomechanical theoretical substrate), any temporary local and neurological tissue changes are sought that are perceived as positive-ameliorative-pleasant (even if it worsens the pain stimuli but that pain stimulus is perceived as “good”) by the operator and patient, both in touch-stimulated body regions and in remote areas [19]. In addition, the re-execution of the physical examination test (semeiotic test consistent with the reason for consultation and medical diagnosis), functional physical examination (evaluation of the regulatory functions associated with SD), the daily gesture (perceived by the patient as correlated with the reason for consultation, i.e., familiar symptoms and comparable signs), must end up into a better outcome in order to drive a decision of a particular approach to be selected [5,8].

Moving into the pragmatic procedure, we can summarize that the type of touch that is perceived as pleasant by the patient should be considered for the selection of the therapeutic approach (e.g., direct, indirect, combined methods: The terms direct-indirect are used to define a touch of approach-departure from a restrictive tissue barrier perceived by the operator during the execution of tests and techniques. The words do not refer to barriers to be corrected, nor to correlations between biomechanical misalignments and health, not to specific effects of detailed mechanical-vector-based-techniques) [19]. Positive exteroceptive stimuli (such as touch) are likely to activate the interoceptive areas of the brain, modulating anxiety and pain response [19]. Conversely, negative experiences can increase the behaviour and symptoms of the disease [19]. For example, improving the active range of motion or pain, as well as enhancing the individual perception of the agency free from uncertainty after a specific type of touch dispensed by the osteopath, could help the osteopath and the patient to select the most appropriate approach [19]. In light of these hypotheses, a patient’s pleasant reactivity to rapid compressive touch could drive the selection of high-velocity low-amplitude techniques, recoil techniques, and the patient’s active approaches, such as rapid stimulation exercises (e.g., using foam roll and rebound elasticity movement). Conversely, positive responsiveness to osteopathic slow-touch administered along the tangential vectors could drive the choice of indirect myofascial release and active melting stretch approaches [19]. 

**Figure 3:**
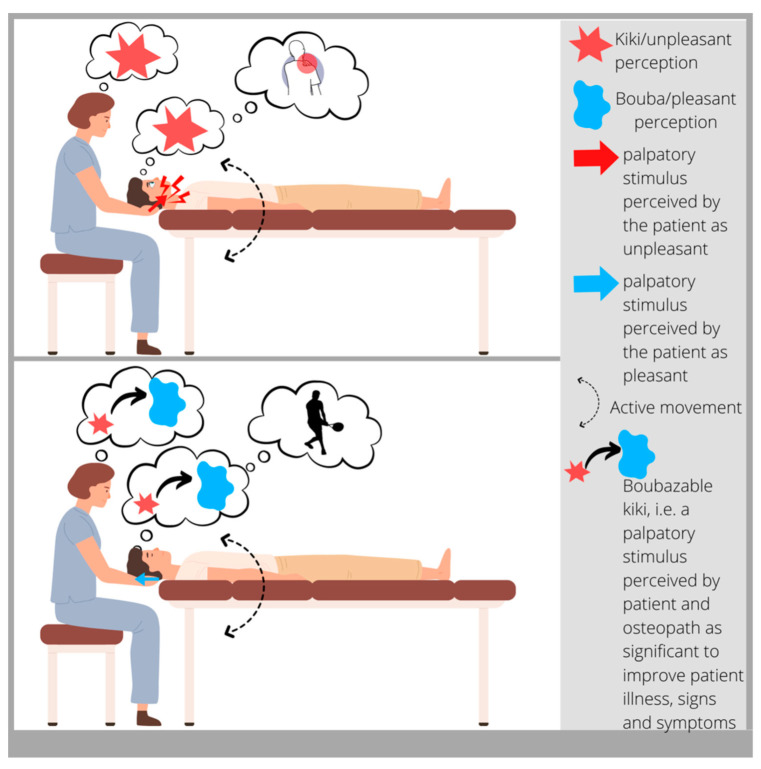
SFCT in the Neuroaesthetic Enactive perspective. The SFCT aims to produce a pleasant surprise (in the figure: patient perception of improved pain and motion of the right upper limb) and a significant prediction error that will defy prior assumptions and update the brain’s generative model (in the figure: I can go back playing tennis) by a nonverbal exchange between the patient and the osteopath, supported by narrative interactions.

The SFCT is not to be intended as a clinical tool for the diagnosis of organic disease or pre-subclinical conditions, as in some cases has been hypothesized for manual muscle testing [74,75]. Conversely, it is thought to be a nonverbal exchange between the patient and the osteopath (supported by narrative interactions), intended to produce a pleasant surprise and a significant prediction error that will defy prior assumptions and update the brain’s generative model (Figure 3).

#### What If the SFCT Is Negative?

According to the patient case history and functional physical examination findings, the osteopaths select systemic maximalist approaches that involve the use of general types of touch and global body positioning to support regulatory functions [5] (e.g., pumping type of touch to improve lymphatic flow, typically used in lymphatic pump techniques [76]; or a type of touch which has been proposed to have an interoceptive value like fascial unwinding [77]; mindfulness-based and meditative-ideomotor movements to balance autonomic nervous system pathway. Systemic/functional (i.e., aimed at promoting a specific function) approaches (e.g., general fascial patterns approach; CV-4 technique; lymphatic pump technique, etc.) might be of use in combination with active patient approaches (i.e., mindfulness-based strategies, experiential bodywork [18,78] and specific exercises) in patients experiencing a high allostatic load sustained by a central sensitisation state and interoceptive failures (i.e., cases in which SFCT might fail to highlight connections between structures and functions). The administration of this type of treatment aims to obtain greater patient adherence, improve the functionality of the patient’s regulation systems, and have positive results with SFCT in the following encounters. Consequently, a personalized approach could be administered (i.e., minimalist approaches based on SFCT positive results). The best available evidence and clinical experience used in a tailored fashion within the specific clinical case (according to the neuroaesthetics paradigm) are always to be considered during the entire clinical reasoning process.

In appendix A, the application of SFCT has been described in detail through Simplified clinical scenarios (see Appendix A. Simplified Clinical Scenarios).

**Figure 4:**
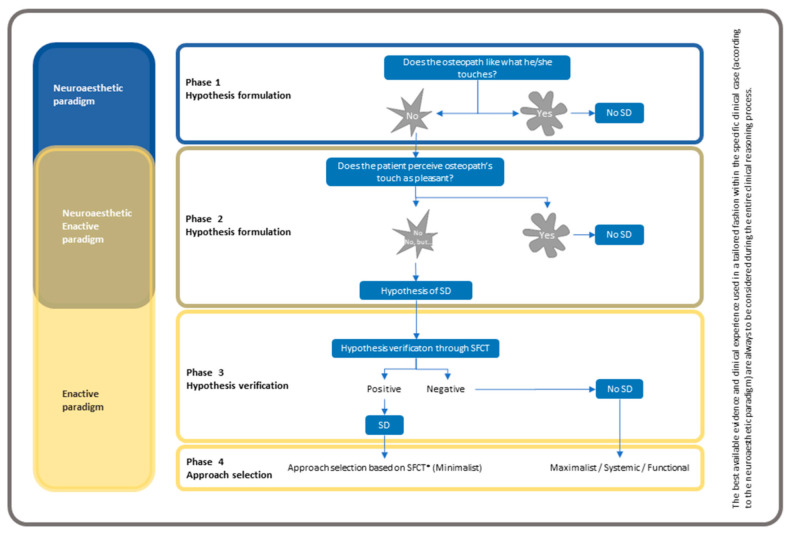
SFCT algorithm: hypothesis formulation, validation phases and approach selection.

A graphical representation of the decision-making algorithm is presented in Figure 4.

## 3. Discussion

The present perspective article is a revised clinical interpretation of the concept of SD, in which patients and osteopaths are both active parts of a shared sense-making process. We provided a renovated framework to describe an enactive process [11] based on neuroaesthetic experiences that occurred during osteopathic encounters [19]. The diagnostic procedure, not just the therapeutic intervention, is intended as a patient–osteopath verbal and non-verbal dialogue to cause a positive surprise and a high prediction error that will violate existing predictions and update the brain’s generative model [11]. The proposed shared sense-decision-making process is based on proximity, and non-verbal approaches, particularly touch and can be reinforced by verbal communication with the person or caregiver. We speculate that sharing a pleasant perception resulting from a personalized selected type of executed touch (not another) in a patient body region (not another) can increase perfusion in the medial-orbital-frontal cortex, conveying non-specific effects, namely placebo. This process accompanies the patient in giving meaning to the presented disorder, and discomfort, to the progression of his health and performance. It also supports the process of giving meaning to the proposed care approach; for example, since osteopathic approaches can involve hands-on techniques by touching remote areas compared to those in which the symptom is perceived, those can be difficult to understand following verbal explanations. Furthermore, the proposed strategy makes it possible to avoid the patient, not having well understood the nature of the proposed treatment, and the reasons for a “novel” approach to be proposed, may have unpleasant perceptions, thoughts, negative beliefs, expectations, and favour nocebo-related effects during all phases of the therapeutic encounter [79]. The presented model is consensual with the principles of person-centred care for musculoskeletal pain management [80]. Putting person-centred care principles into practice could favour the establishment of meaningful connections by using the body as a pivot point [80]. Assisting the patient’s connection to the body and employing touch to close a gap are all components of this method, implemented to clarify issues and related treatments [80]. In this approach, the “bio” in biopsychosocial performs a function that goes beyond diagnosis and treatment; it becomes a crucial element in creating connections and has been cited by patients as crucial for a fruitful clinical encounter [80]. 

### 3.1. Strengths and Limitations

This proposal represents a practical tool for practitioners to integrate patients into clinical decision-making and it updates with contemporary rationales the philosophical roots of the profession highlighting its specificity. Moreover, it helps to interpret the existing literature on the reliability of manual assessment in a new light, with a focus on the reciprocal perceptions of both patient and osteopath rather than on the value of inter and intra-rater reliability. This also helps to move from a sole tissue-based rationale, in which the assumption is that there is something “wrong” in the tissues that it is having a clinical impact on the subject adaptive capabilities (which is not always the case, especially in chronic conditions or in centrally sensitized patients), to a vision in which the SD is a communicative entry key to the patient’s central nervous system. In this perspective, the fact that different practitioners find with the help of the patient this key in different body areas is, in absolute terms, a “non-problem”.

This proposal presents some possible limitations. One concern is about the functional tests used during the SFCT. Not all functional tests are eligible to be selected during the SFCT. The test’s ability to act as a decision driver (i.e., gives immediate response, e.g., one-legged standing test [OLST]) or as a decision moderator (i.e., cannot give immediate response and it needs time to become negative, e.g., erythrocyte sedimentation rate) is to be considered. Tests that act as decision moderators are useful to plan the follow-up and monitor the process over time, but they are not employable during SFCT because the SFCT is based on immediate response. Decision driver tests are eligible to be selected for SFCT, but they need to be critically apprised to understand if they are practically employable during SFCT. For example, the OLST [81] is used both to assess the postural control capability of a subject (as a more generic test) and to assess ankle functional instability (as a more specific test). The tested subject needs to keep the equilibrium on one leg. It is easily imaginable that applying external forces (like those needed during SFCT) might alter the test execution and therefore invalidate the test results. It makes the OSLT challenging to use in association with SFCT. Another aspect to be discussed concerns the use of the functional tests used during the SFCT in infants and children. There are results from a qualitative study reporting the osteopaths’ use of general movements and neural-motor developmental stages as decision-making moderators, which may be considered useful as decision drivers [82]. Moreover, experienced osteopaths reported paying attention to allostatic and stress processes in neonatal and pediatric clinical practice [82]. As nurses and other neonatal care professionals, osteopaths could implement neonatal physiological and behavioural stress assessment [83]. For example, changes in infant state and colour, motor disorganization, and autonomic signs could represent decision drivers to evaluate overstimulating or pleasant stimuli during the encounter [83]. For instance, Tarantino and colleagues considered the facial expression, the existence of reflex movements, sobbing, or a quick rise in heart rate, and the response to slight compression to evaluate the tenderness status, as one of the parameters to be considered in the assessment of SD [84].

One of the main aims of the presented paradigm is its ability to transform the patient into an active element, with the autonomy to influence the effects of treatment and the capacity to improve their illness. The declared goal result is easily doable with people with healthy internal locus who believe that their behaviour influences health [85]. Conversely, the neuroaesthetic-enactive paradigm could be challenging to implement with patients with external health locus of control. They are usually not collaborative, delegate decisions to practitioners, and do not want to be involved in shared decision-making because they prefer to delegate to other “powerful” people [85]. Therefore, osteopaths and other health professionals should consider psychosocial features and personality traits when planning and implementing patients’ treatment [85]. 

There are open questions that might be addressed in future studies on the fitness of different functional tests to the scope. Another limitation is represented by the lack of experimental evidence (in this specific context) of the proposed neurobiological mechanism proposed in the rationale (i.e., neuroaesthetics and active inference). There are several studies addressing the neurobiological processes underlying neuroaesthetics and active inference, but to the best of the authors’ knowledge, the experimental settings were never specifically the described type of interaction between osteopaths and patients. Future research might fill this gap and provide experimental evidence to support (or refute) the present proposal. 

### 3.2. Future Directions

The present report delineates the future directions for the reconceptualization of the SD concept and the application of the Neuroaesthetic Enactive Paradigm in osteopathic practice. The first step of the road map could be to discuss evidence-informed recommendations to assist operators and patients in a multidimensional domain through a consensus conference: a valuable tool to address agreements about central debated concepts into communities of health professionals, and produce useful recommendations for guiding clinical practice [86]. The involvement of the international osteopathic community of practice could help explore the state of use of the paradigm and highlights its potential to improve outcomes, catch the peculiarities and problems, and eventually expresses ideas on how to improve its distribution to both students and practitioners. 

Moreover, the opportunity to consolidate a decision-making process and to share it with the community of practice and the scientific community would give the opportunity to strengthen future experimental clinical studies. To date, to the best of the authors’ knowledge, most of the randomized controlled trials in the osteopathic field relied on a “black box” type of intervention. Adopting a shared algorithm to guide the decision-making in the clinical experimental setting would help to look into the “black box” and to strengthen the internal and external validity of future clinical research.

## 4. Conclusions

The present perspective article illustrates a proposal to overcome the debate around SD, rather than dismissing the concept. We proposed the integration of tradition, evidence, artistry, and clinical experience, through a pragmatic application of the (en)active inference and neuroaesthetic theoretical frameworks. We claim that the presented renovated concept of SD, integrating technical rationality and professional artistry clinical experience [87], can be a useful tool to achieve a creative, evidence-informed, and shared sense-making process between patients and osteopaths.

“*Researchers, whether they are scientists or artists, brave the unknown by starting from what is known. Nowadays, the key distinctions may not end up being seen to be between creative and formulaic thinking, rather than between artists and scientists, but between those who can accept doubt and those who cannot” [88].*

## Figures and Tables

**Figure A1:**
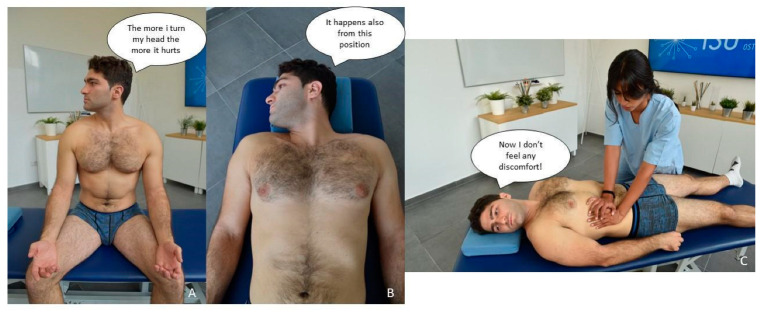
Clinical scenario 1.

**Figure A2:**
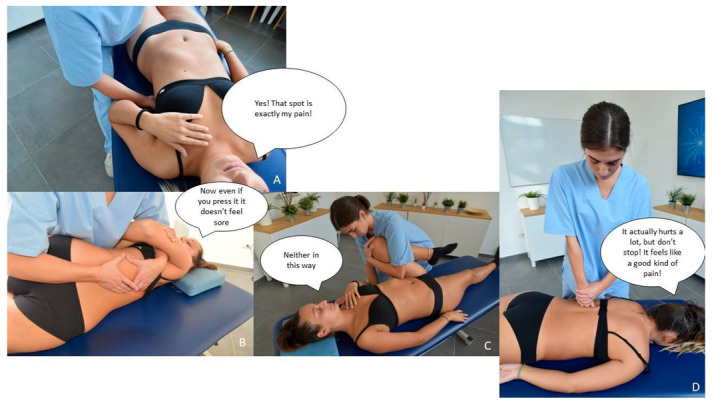
Clinical scenario 2.

**Figure A3:**
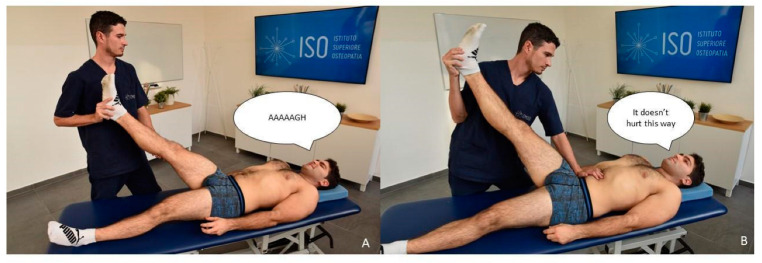
Clinical scenario 3.

**Figure A4:**
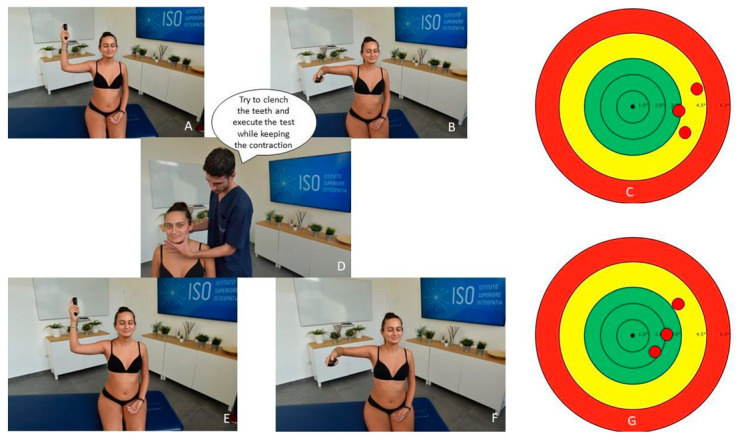
Clinical scenario 4.

**Figure A5:**
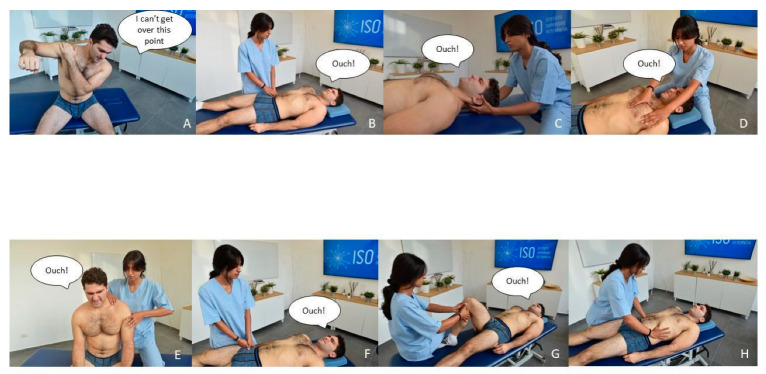
Clinical scenario 5.

## Data Availability

Not applicable.

## Supplementary Materials

The following supporting information can be downloaded at: https://www.mdpi.com/article/10.3390/healthcare11040479/s1, Table S1: Somatic dysfunction, historical overview. References [89,90,91,92,93,94,95,96,97,98,99,100,101,102,103,104,105,106,107,108,109,110,111,112,113,114,115,116,117,118,119,120,121,122,123,124,125,126,127,128,129,130,131,132,133,134,135,136,137,138,139,140,141] are cited in the supplementary materials.

## Appendix A. Simplified Clinical Scenarios

The following clinical vignettes are simplified clinical scenarios that assume that each situation has already been assessed in terms of biomedical (i.e., differential diagnosis; pain neurophysiological mechanism following IASP classification, etc.) and biopsychosocial elements (e.g., maladaptive cognitions, emotional dysregulation, socio-environmental context, etc.) Therefore, all the scenarios start from the osteopathic examination to show how the decision-making process concerning the selection of osteopathic personalized hands-on/off approaches takes place. Moreover, the number of palpatory findings has been shortened to ease the reading compared to the ones usually encountered in daily clinical practice. Lastly, the scenarios represent a single treatment without describing the follow-up selection process, which is not the scope of the present perspective article.

*Clinical scenario n. 1* (Figure A1)

Reason for consultation: mechanical neck pain

Comparable sign: pain on the base of the neck elicited during right rotation of the head from a sitting and supine position (Figure A1A,B).

Emergent palpatory findings:

- Right hypochondrium.
  - Osteopath perceptions: stiff tissue, restriction barrier in upward-right direction;
  - Patient perceptions: discomfort with the palpation especially during upward-right compression.
- Mid-thoracic spine trait (T4–T6).
  - Osteopath perceptions: the osteopath observed a reduction in the thoracic spine kyphosis during the active flexion movement during the standing position; stringiness on the perivertebral soft tissues;
  - Patient perceptions: the patient reports feeling “nothing relevant” during the active movement and the palpation.
- Left temporal area of the head, suboccipital right area.
  - Osteopath perception: slightly swelling tissue in the area, reduced gliding capacity of the skin;
  - Patient perception: the patient reports feeling “nothing relevant” during the palpation and the gliding.

SFCT (employing comparable sign): the osteopath, with the patient in a supine position, applies slow and progressive compression on the right hypochondrium and asks the patient to reiterate the provocation test (i.e., comparable sign). The test is positive since during a specific compression of the right hypochondrium with upward-right parameters the patient refers to not feel any discomfort during the active movement of the right rotation of the head (Figure A1C) All t other types of touch (e.g., other directions of compression; traction, torsion, etc.) provided in the same area did not negativize the provocation test.

Selected osteopathic approach: direct myofascial release (abdominal region SD, right hypochondrium) with the patient in a supine position; direction of the manual input: upward right.

*Clinical scenario n. 2* (Figure A2)

Reason for consultation: bilateral dorsal resting pain (occurred after the patient collided forcefully playing basketball). The pain is not perceived during active or passive movement.

Familiar symptom: hyperalgesia on a specific spot in the low dorsal area (Figure A2A).

Emergent palpatory findings:

- Left Hip.
  - Osteopath perceptions: bogginess in the anterior region of the hip (i.e., hip socket face), limitation on the passive hip extension and internal rotation;
  - Patient perception: altered sensitivity during palpation, feelings of tension during passive extension.
- Lumbar area.
  - Osteopath perceptions: generally stiff tissue on the entire lumbar area, limitation to the active and passive movement during right trunk rotation;
  - Patient perceptions: generalized discomfort during lumbar palpation, feels limitation during active and passive movement.
- Thoracic area.
  - Osteopath perceptions: altered breathing pattern, left rib cage seems to have a greater excursion than the right one; right rib cage feels less elastic when compressed;
  - Patient perceptions: nothing in particular during the assessment of the area.
- Tender point on the dorsal area.
  - Osteopath perceptions: a stiff spot on left paraspinal soft tissue (approximately between T8–T10);
  - Patient perceptions: recognize the painful sensation elicited by the manual compression on the spot as “his pain”.

SFCT (employing familiar symptoms):

- The osteopath sets the patient in a side roll position with right lumbar rotation rapid compressions until the perceived restrictive barrier is engaged; monitoring and asking for feedback from the patient about the tender point (i.e., familiar symptom; provocation test). The test is positive since the patient reports diminished pain the more the osteopath comes close to the restrictive barrier (Figure A2B);
- The osteopath, with the patient in a supine position, applies a progressive passive flexion movement of the left hip until the physiologic barrier is perceived; continuously monitoring and asking for feedback from the patient about the tender point (i.e., familiar symptom; provocation test). The test is positive since, during a passive left hip slow and progressive flexion and external rotation, the patient improves hyperalgesia on the specific spot perceived in the low dorsal area (Figure A2C). All the other types of touch (e.g., other passive movement or type of touch; positioning in the direction of the restricted barrier, compression, traction, torsion, release after muscle contraction, etc.) provided in the same area did not negativize the provocation test;
- The osteopath applies a contact force by a progressive touch on the tender point asking for feedback from the patient about the tender point (i.e., familiar symptom; provocation test). The patient answers that it hurts but “it feels like a good pain”. The test is therefore considered positive (Figure A2D).

Selected osteopathic approaches:

1. Facilitated positional release (lower extremities SD, Left hip) with the patient in a supine position; direction of the manual input and passive movements: combined indirect movements of the left hip (flexion and rotation);
2. High velocity, low amplitude technique (lumbar region SD) with the patient in a side-roll position; direction of the manual input and passive movements: right rotation (and accessory planes of motion) is implemented until the restrictive barrier is engaged, and then a rapid force of brief duration driving a short distance is applied within the perceived anatomic range of motion;
3. Inhibitory pressure technique (thoracic region SD) with the patient in a prone position.

*Clinical scenario n. 3* (Figure A3)

Reason for consultation: lumbar bilateral pain radiated on the posterior area of the right gluteus and the tight. MRI negative for radicular impairments. The orthopaedic specialist referred the patient to osteopathic care.

Semeiotic test: positive lasegue test (Figure A3A)

Emergent palpatory findings:

- Right iliac abdominal region.
  - Osteopath perception: swelling, bloating, lowered compliance to compression;
  - Patient perception: discomfort during the palpation, reports referred pain to the lumbar area.
- Right shoulder.
  - Osteopath perception: observed scapula dyskinesia during the active test, limitation to the gliding of the subscapular surface during the passive test;
  - Patient perception: nothing relevant.
- Sternal area.
  - Osteopath perception: stiffness of tissues, impaired elastic response to compression;
  - Patient perception: nothing relevant.
- Dorso-lumbar area.
  - Osteopath perception: passive range of motion perceived as limited, perivertebral soft tissue generally stiff.
  - Patient perception: the zone feels stiff but “normal stiff”.

SFCT (employing semeiotic test related to the chief complaint): the osteopath, with the patient in a supine position, applies an oscillatory manual force to the right iliac abdominal region and executes at the same time the Lasegue test (semeiotic test/provocation test). The SFCT is positive since the Lasegue test is frankly negative (Figure A3B). All the other types of touch (e.g., other passive movement or type of touch; positioning in the direction of the restricted barrier, compression, traction, torsion, release after muscle contraction, etc.) provided in the same area did not negativize the provocation test.

Selected osteopathic approach: facilitated oscillatory release (Abdominal region SD; right iliac abdominal region) with the patient in a supine position.

*Clinical scenario n. 4* (Figure A4)

Reason for consultation: Asymptomatic patient seeks consultation as a professional javelin thrower to improve throwing performance.

Functional physical examination: positive shoulder joint position sense error (JPSE) test, the first tests are worse than the last in all tested movements (Figure A4A–C).

Emergent palpatory findings:

- Bilateral temporal area.
  - Osteopath perception: ropiness of soft tissues of the area, reduction of the gliding of the skin on subcutaneous tissues;
  - Patient perception: referred pain to the right masseterine area during digital pressure of the temporal area, pin-like pain during gliding.
- Right masseter area.
  - Osteopath perception: stiffness of the soft tissue of the masseterine area; altered mandibular kinematic during mouth opening and closing;
  - Patient perception: referred pain to the right temporal area during digital pressure of the masseterine area; general discomfort on the temporomandibular joint area during last degrees of mouth opening; feels asymmetric pattern during the motion of the jaw.
- Left ankle area.
  - Osteopath perception: slight sponginess of external peri-malleolar area, limited range of motion in dorsiflexion;
  - Patient perception: nothing relevant.

SFCT (employing functional objective examination test): The osteopath asks for an isometric contraction of occlusal muscles, with the patient in a sitting position and asks the patient to execute, at the same time, the JPSE test (functional objective examination). The SFCT is positive since the JPSE test is frankly negative (Figure A4D–G). All the other types of touch (e.g., other passive movement or types of touch; positioning in the direction of the restricted barrier, compression, traction, torsion, etc.) provided in the same area did not negativize the JPSE test.

Selected osteopathic approach: muscle energy technique of the occlusal muscles.

*Clinical scenario n. 5* (Figure A5)

Reason for consultation: intermittent and generalized myalgias and arthralgias without true inflammatory arthritis, chest heaviness, and breathing difficulty, with additional symptoms including fatigue, brain fog, and consequently, loss of agency.

Medical diagnosis: Long COVID. Treatment: nutrition and rehabilitation programs; referral to an osteopath for a consultation as a hands-on therapist, and as a health coach with a tertiary prevention aim.

- Familiar Symptom: reduced range of motion of flexion and abduction of the right shoulder (Figure A5A);
- Comparable sign: symmetrical tender points located both above and below the waist around the neck, chest, shoulders, hips, and knees (Severe pain, 7 on a 0–10 pain scale) (Figure A5B–G);
- Functional physical examination: Manual assessment of respiratory motion showed reduced lower rib cage/abdomen motion during breathing [142] (Figure A5H);
- Manual assessment tests of central sensitization [143]: positive (hypersensitivity to a stimulus was demonstrated at both symptomatic and distant sites).

Emergent palpatory findings:

- Occipital area
  - Osteopath perception: augmented stiffness in the area, reduced gliding capacity of the skin;
  - Patient perception: the patient reports feeling “dizzy” during the palpation of the occipital area.
- Thoracic area
  - Osteopath perceptions: altered breathing rate (rate of 12 breaths per minute), augmented stiffness in the whole rib cage, decreased range of motion in the right rib cage;
  - Patient perceptions: nothing in particular during the assessment of the area.
- Generalized fascial pattern (spiral and back functional lines—myofascial chains)
  - Osteopath perception: preferred direction of motion throughout the spiral and functional back lines [144];
  - Patient perception: perception of passive or active positioning as antalgic postures (alternating progressive slow positioning to maximize tension, stretch, and then release).

SFCT: all negative; no one of the palpatory findings were relevant for patient responsiveness.

Selected osteopathic approach: because of the negative findings emergent from SFCT, osteopaths considered the finding of the research conducted in clinical contexts similar to the patient presentation, in order to select the best method to address the patient’s symptoms. The approach is contextualized by evaluating its applicability to the individual patient, preferring the osteopathic approaches selected in the osteopath/patient dyadic relationship. The utility of osteopathic care in a patient population with respiratory tract infections sequelae was described in prior investigations [145]. Moreover, it has been reported that several osteopathic approaches could intercede with sensitization states, at all levels, using interoceptive pathways [146] to address biopsychosocial factors that perpetuate patients’ chronicity [147]. According to the patient’s perceptions, the more acceptable approaches were selected [145]. The patient preferred to be positioned in the spiral and functional back lines “antalgic” postures [3,144] while an abdominal lymphatic pump, thoracic diaphragm release, and rib raising techniques were administered [145]. To help the patient in the reconceptualization of pain and symptoms, patient active osteopathic approaches were implemented [18]. It was preferred a combination of hands-on and hands-off techniques, especially interoceptive touch-based osteopathic strategies, mindfulness-based exercises, mental imagery, etc. [18].
