# Supplementary material for: Reconceptualizing Somatic Dysfunction in the Light of a Neuroaesthetic Enactive Paradigm
Source: Healthcare (Basel). 2023 Feb 7;11(4):479. doi: 10.3390/healthcare11040479 (PMC9957393; doi:10.3390/healthcare11040479)
Supplement: Supplementary file 1 [file healthcare-11-00479-s001.zip › healthcare-2028202-Supplementary Materials.pdf]

healthcare

MDPI

Supplementary Materials:

Table S1. Somatic dysfunction, historical overview and ongoing debate.

1.1 Somatic Dysfunction: history, evolution, definition and research. Somatic dysfunction (SD) is a key element of osteopathic practice [1,2]. SD was originally mentioned as Osteopathic Lesion (OL) by A.T. Still [3]. The OL was considered an alteration of structure/function interdependence whose treatment might have helped the body to adapt from the "abnormal" to the "normal" state, to favour the body's ability to protect itself against illness, and regains health by its inherent abilities [4]. Osteopathic early writings were characterized by regional anatomical approaches to the person [4]. In Still's idea, the body regions (originally mentioned as five: head, neck, chest, spine, pelvis and limbs) needed to work as a whole during the body motion. The regions were linked to various functions (e.g., body fluids flow, neural transmission, excretory processes, etc.) [4]. The pioneering osteopaths considered how environmental factors, infectious agents, nutritional issues, and emotional variables could facilitate the occurrence of OL, and, therefore, influence health [5]. Following Still, Takser [6] and Hulet [7] considered the OL as any structural change that negatively affects tissue and self-regulative systems' functionality. They highlighted that there may be structural changes noticeable on palpation, but which do not modify self-regulative functionality and, consequently, are not considered OL. The OL concept evolved into two different branches. On the one hand, there were osteopaths such as Littlejohn, Fryette, and Downing [8], that developed the idea of an "environmental lesion", or "total lesion", and "greater osteopathic lesion complex" [3]. The body was considered an organism, rather than a mechanism. Function and structure changes might have been associated with mental and psychological states, as well as nervous, circulatory, secretory, and excretory systems regulation [3]. The mentioned adaptive health processes were considered to favour generalized disturbances, or regional symptoms close to and distant from tissues involved in the regulative processes [9]. On the other hand, it was developed a more mechanical-oriented concept in which osteopaths referred to the OL as a result of trauma initiating the somatic-visceral reflex, and the effects of repeated trauma, usually on a spinal joint, with associated changes in ligaments and soft tissue structure [10]. While in early 1900 the adoption of the term OL was used to describe a primary factor influencing body-mind functionality and health, or as a biomechanical issue that causes illness, in the mid-60 a small group of intercollegiate recognized the risk of a misinterpretation of the term "lesion", used in biomedicine to define "an abnormal change in the structure of an organ or part due to injury or disease" [11]. The term OL was dismissed and changed in SD in 1968 [3] by a working group of the Educational Council on Osteopathic Principles (ECOP), led by Ira Rumney, and Norm Larson [4]. The Hospital Assistance Committee of the Academy of Applied Osteopathy, chaired by Ira Rumney, DO, developed definitions for osteopathic diagnosis and treatment, including SD, for inclusion in the Hospital International Classification of Disease (ICD). To date, SD is still listed in the present version of the ICD [12].<br>Nowadays the Glossary of Osteopathic terminology defines SD as an "Impaired or altered function of related components of the body framework system: skeletal, arthrodial and myofascial structures, and their related vascular, lymphatic and neural elements. It is characterized by positional asymmetry, restricted range of motion, tissue texture abnormalities, and/or tenderness" [13]. The latter listed parameters fall under the acronym of TART and have been referred to as the "diagnostic criteria" for SD [13]. Following these conceptual developments, osteopathic basic research in its development over time has focussed on the mechanisms underlying SD features by exploring tissue-related neurological interactions, rather than exploring osteopathic palpatory findings related to patient-environment (or context) interactions (i.e., functions). Marion Clark [14] suggested that SD was a result of connective tissue inflammation. Louisa Burns [15] investigated the dysfunctional visceral and somatic reflexes associated with SD, reporting vascular changes in the fascial tissues, signs of inflammation, exudation and altered tissue texture associated with vertebral SD [16]. Cole [17], Korr [18] and Denslow and colleagues [19] observed the presence of aberrant somatic-visceral reflexes as the basis for the existence of facilitated areas in the spine that corresponded with the palpable features of the SD. Research findings from Hix [20], Beal [21], Kelso and colleagues [22], Van Buskirk [23], Fryer and colleagues [24–26], were organized into the nociceptive model. In particular, Fryer's proposal [27] arises from the concept that tissue damage causes inflammation with consequent neurogenic inflammation (nociceptors activation) brought on by dorsal root reflexes. The inflammation process might generate confounding factors for palpation of tissue texture abnormalities and the tenderness could be considered a consequence of central or peripheral sensitization, which occurs as a result of increased excitability of neurons in the central nociceptive pathways [27,28]. Tozzi [29] presented a conceptual shift from the nociceptive model to a neuro-fasciagenic model that integrated neural influences into a multifactorial and multidimensional interpretation of dysfunctional processes mediated by the fascia. Verzella and colleagues [30], in their recent review, proposed a new model relating to the characteristics of SD and its clinical implications by linking it to body fluids in the extracellular matrix dynamics, and low-grade inflammation [30]. Other authors proposed a revision of the concept underlying the biomechanical model and its application [31]. The updated model does not aim to restore the ideal posture by correcting a mechanical misalignment [31]. The authors proposed SD as a tool to personalize manipulative and patient-active

approaches [32], to promote interoceptive and proprioceptive integration and to optimize individual responses to the existing allostatic load [33] (i.e., biological and psychological functions [34]). In light of the evolution of the concept, it can't be said that the SD is to be considered a source of nocebo [35], but it is a misinterpretation of the notion that is based on mechanistic spine-centric models of health and disease that represent the origin of potential nocebic outcomes.

1.2. Somatic Dysfunction: an ongoing debate. Despite the interpretative theoretical models for SD proposed in osteopathic basic research, the clinical relevance of SD is strongly debated [27,36–39]. The discussion around SD shifted entirely to the concept that SD relies on a simple cause-effect model of osteopathic care [39]. The cause-effect model applied to SD was focused on detecting and resolving objective structural signs at the origin of the patient's symptoms [39]. The authors who criticized that model advised osteopaths to be more focused on the importance of other aspects of care, such as practitioner-person interactions [39]. Other authors claimed that scientific findings had not clarified the relationship between somatic dysfunction and health status [38], therefore, despite the biological plausibility of SD its clinical relevance remained uncertain. Moreover, they underlined that there is poor diagnostic reliability and unclear validity for SD [38,40–42]. On top of that, even if clinical phenomena associated with somatic dysfunction, such as TART, may be biologically plausible, the idea fails to integrate social and psychological aspects [27,39], neglecting many valuable avenues for patient education strategies [27,39]. The authors suggested the academics involved in the curriculum design processes, explicitly requesting them to be more focused on the prevention and treatment of non-communicable disease by modifiable determinants of health (i.e., diet, physical activity, sleep, and stress reduction) rather than SD [38,39]. Additionally, the use of terminology and special diagnostic entities only detectable to the practitioner could potentially reinforce unhelpful patient perceptions (i.e., maladaptive cognitions) about the nature of symptoms and become an obstacle to recovery (i.e., nocebo effect) [38,39]. For example, it seems that osteopathic manual practitioners in Canada continue to use the term osteopathic lesion [43]. The adoption and misappropriation of a biomedical expression and the use of the same curative reasoning processes as in biomedicine can favour potential iatrogenic consequences for patients [43].

All the studies mentioned above relied mainly on the critics of TART parameters trying to link a palpatory finding to a clinical condition (foraging a cause-effect idea of SD) and assessing the reliability of palpation as a standalone diagnostic procedure [40–42]. In this process, SD became a synonym for TART parameters. However, in all the definitions of SD through time, the only fil-rouge was represented by the presence of an "altered function" that needed to be assessed in association with the body structures (i.e., body regions). The sole presence of one or more positive palpatory findings, indeed, was not considered a satisfactory condition to address a clinical entity such as SD. On these bases, the critics moved to SD to date are only partially related to SD, but for the most are addressed to a misinterpretation of SD. Some authors [44] proposed new interpretative models of SD in osteopathic clinical practice, drawing on complexity literature. Hence, SD was not considered in a cause-effect relationship with the patient clinical presentation (i.e., Cynefin framework simple domain), but the relationship between SD and the clinical presentation was considered a complex relationship whose properties were assessable only in a retrospective way through emergent patterns (i.e., Cynefin framework complex domain) [44]. Those emergent patterns implied an active role of the patient in the assessment process that was completely absent in the sole TART evaluation. The adoption of a shared decision-making procedure [45], in which patient's responsiveness, body narratives and perceptions are taken into high consideration, helps to move from SD, as a biomechanical tissue impairment, and its maladaptive cognition/nocebo correlates, to comprehensive patient-centred care, that includes both biological and psychosocial aspects of the person's life, as well as their impact on patient clinical presentation and health. Some recent proposals for interpreting SD tried to refocus the definition of SD by including aspects related to function. Bergna and colleagues [46] presented a new perspective for SD in Osteopathy: the variability model. The authors assumed that movement variability could be related to "body adaptability"; it would allow interpreting SD clinical signs as an attempt by the body to maintain health [46]. In their proposal, particular emphasis is given to biomechanics and parameters only assessable by the practitioner in a non-shared decision-making fashion. However, in the recent application of the variability model in a retrospective cohort study, the authors attempted to test the hypothesis that movement variability impacts the body's adaptability, therefore function [47]. Trained pediatric osteopaths applied the variability model to assess somatic dysfunction in the infant study population assessing the movement variability within the neutral zone, myofascial tissue texture, and tenderness [47]. To evaluate the tenderness status, the reaction during slight compression between the head and sacrum was evaluated using facial expression, the presence of reflex moves, crying, or the sudden increase in heart rate [47]. Lunghi and colleagues [33] have described how tissue changes (i.e., reversible strains) can be consequent to the adaptation processes (i.e., allostatic process) that involve all biopsychological regulation systems, also influencing perception processes (exteroceptive, interoceptive, proprioceptive), body awareness, body image and body pattern [33]. SD has been therefore considered one or more regions where there are reversible elastic strains, which are neurologically active (i.e., can elicit patient responsiveness to touch) and can be used to convey the effects of touch [45] and patient active movements [32]. It follows that the diagnostic evaluation process must necessarily be shared with the patient, also taking into high consideration their perceptions, preferences, responses and verbal and non-verbal narratives [34]. In this viewpoint, the diagnostic nomenclature is in accordance with the one used in the ICD-

11 [12] that mainly focuses on the definition of areas (i.e., body regions) as opposed to the biomechanically oriented terminology used in the definition of the specific body segment where one or more TART parameter are potentially palpable. Implementing the International Classification of Functioning (ICF) [48] in the osteopathic practice for recording problems involving functions and structures of the body, activity limitations and participation restrictions manifested in patients, and relevant environmental factors will help osteopaths enforce a common language with other health professionals [49,50]. Indeed, osteopaths participants in a recently published qualitative research [51] declared that there is a need for osteopathic professionals to employ the ICF to improve interprofessional practice and multidisciplinary patient-centered approach. The vast majority of osteopathic scientific reports mentioned SD as the clinical entity approached by the practitioner in the experimental phase [52], and despite the ongoing debate, SD keeps a key role in osteopathic clinical practice and research [52]. Literature provides different insights into the clinical usefulness of SD for osteopathic practice. The specific osteopathic treatment, intended to normalize SD, seems to show a better benefit than the simple non-specific (i.e., placebo) touch. The general effects of touch may contribute to the overall effectiveness of Osteopathic Manipulative Treatment (OMT). In fact, a recent crossover investigation [53] demonstrated that OMT could increase the corticospinal excitability caused by transcranial magnetic stimulation on volunteers with SD, but not sham treatment. In a different study [54], asymptomatic young volunteers who received OMT but not sham therapy experienced changes in their resting cerebral perfusion as determined by magnetic resonance arterial spin labelling. Functional magnetic resonance imaging was used in a study by Cerritelli and colleagues [55] to examine the effects of OMT and gentle touch on people with chronic lumbar pain. The findings showed that only OMT improved behaviour in an interoceptive awareness test and elicited a discrete and specific response in brain regions connected to interoception (i.e., bilateral insula, anterior cingulate cortex, left striatum, and right middle frontal gyrus). Therefore, the authors came to the following conclusion: "The post-treatment resting-state contains osteopathic-related effects that may be linked to the special touch-based nature of osteopathy". Beyond the improvement in pain-related function [56–58], OMT has been linked to a number of additional subjective and behavioural changes, including the promotion of confidence during movements without pain [57–59], decreased anxiety, and improved global body perception in female participants [60]. Osteopathic touch, with its inherent non-specific characteristics, also performs psychosocial functions. It is a method of communicating [61–63], of reassuring the patient [58,59,63], and as a result, it is a crucial component of the patient-physician relationship with health as its primary objective [62]. Individual psychological elements, such as expectations, and past experiences have an impact on how the patient feels about the practitioner's touch, which in turn can vary the therapeutic outcome [64]. Physical input and cognitive state exhibit functional convergence in the insula; hence, combining bottom-up touch-based and top-down mindfulness-based techniques may improve outcomes in the treatment of interoceptive deficit-related body-mind disorders [64].

1. Consorti, G.; Basile, F.; Pugliese, L.; Petracca, M. Interrater Reliability of Osteopathic Sacral Palpatory Diagnostic Tests Among Osteopathy Students. J Am Osteopath Assoc 2018, 118, 637–644, doi:10.7556/jaoa.2018.132.
2. Sciomachen, P.; Arienti, C.; Bergna, A.; Castagna, C.; Consorti, G.; Lotti, A.; Lunghi, C.; Tramontano, M.; Longobardi, M. Core Competencies in Osteopathy: Italian Register of Osteopaths Proposal. International Journal of Osteopathic Medicine 2018, 27, 1–5, doi:10.1016/j.ijosm.2018.02.001.
3. Liem, T. A.T. Still's Osteopathic Lesion Theory and Evidence-Based Models Supporting the Emerged Concept of Somatic Dysfunction. J Am Osteopath Assoc 2016, 116, 654–661, doi:10.7556/jaoa.2016.129.
4. Seffinger, M.A.; Hruby, R.; Ph. D. Willard, F.H.; Licciardone, J. Foundations of Osteopathic Medicine: Philosophy, Science, Clinical Applications, and Research; 4° edizione.; Lippincott Williams & Wilkins: Philadelphia, 2019; ISBN 978-1-4963-6832-4.
5. Fryette, H.H. Principles of Osteopathic Technic; American Academy of Osteopathy, 1980;
6. Tasker, D.L. Principles of Osteopathy; Forgotten Books, 2012;
7. Hulett, G.D. A Text Book Of The Principles Of Osteopathy; New Publisher, 2021; ISBN 978-0-344-90983-2.
8. Downing, C.H. Principles and Practice of Osteopathy; Tamor Pierston, 1981; ISBN 978-0-907457-00-8.
9. Baroni, F.; Mancini, D.; Tuscano, S.C.; Scarlata, S.; Lunghi, C.; Cerritelli, F.; Haxton, J. Osteopathic Manipulative Treatment and the Spanish Flu: A Historical Literature Review. J Osteopath Med 2021, 121, 181–190, doi:10.1515/jom-2020-0112.
10. The Osteopathic Lesion. Journal of the American Medical Association 1936, 106, 1417, doi:10.1001/jama.1936.02770160075042.
11. Definition of LESION Available online: https://www.merriam-webster.com/dictionary/lesion (accessed on 20 October 2022).
12. ICD-11 for Mortality and Morbidity Statistics Available online: https://icd.who.int/browse11/l-m/en#/http%3a%2f%2fid.who.int%2ficd%2fentity%2f1652981832 (accessed on 20 January 2022).
13. Giusti, R. Glossary of Osteopathic Terminology, Third Edition; AACOM, 2017;
14. Clark, M.E. Applied Anatomy Designed for the Use of Osteopathic Students and Practitioners as an Aid in the Anatomical Explanation of Disease From an Osteopathic Viewpoint Classic Reprint by Marion Edward Clark: New PAP (2019) | PBShop.Store UK;
15. Burns, L. Viscero-Somatic and Somato-Visceral Spinal Reflexes. J Am Osteopath Assoc 2000, 100, 249–258.

16. Burns, L. Secondary Lesions Following Experimental Lesion of the Second Thoracic Vertebra in Rabbits. J Am Osteopath Assoc 1925, 184–188.
17. Cole, W. The Osteopathic Lesion Syndrome. Acad. Osteopath. Med. 149–178.
18. Korr, I.M. The Neural Basis of the Osteopathic Lesion. J Am Osteopath Assoc 1947, 47, 191–198.
19. Denslow, J.S.; Korr, I.M.; Krems, A.D. Quantitative Studies of Chronic Facilitation in Human Motoneuron Pools. Am J Physiol 1947, 150, 229–238, doi:10.1152/ajplegacy.1947.150.2.229.
20. Hix, E.L. Reflex Viscerosomatic Reference Phenomena. Osteopath. Ann. 4, 496–503.
21. Beal, M.C. Viscerosomatic Reflexes: A Review. J Am Osteopath Assoc 1985, 85, 786–801.
22. Kelso, A.F.; Grant, R.G.; Johnston, W.L. Use of Thermograms to Support Assessment of Somatic Dysfunction or Effects of Osteopathic Manipulative Treatment: Preliminary Report. J Am Osteopath Assoc 1982, 82, 182–188.
23. Van Buskirk, R.L. Nociceptive Reflexes and the Somatic Dysfunction: A Model. J Am Osteopath Assoc 1990, 90, 792–794, 797–809.
24. Fryer, G.; Bird, M.; Robbins, B.; Fossum, C.; Johnson, J.C. Resting Electromyographic Activity of Deep Thoracic Transversospinalis Muscles Identified as Abnormal with Palpation. J Am Osteopath Assoc 2010, 110, 61–68, doi:10.7556/jaoa.2010.110.2.61.
25. Fryer, G.; Morris, T.; Gibbons, P.; Briggs, A. The Electromyographic Activity of Thoracic Paraspinal Muscles Identified as Abnormal with Palpation. J Manipulative Physiol Ther 2006, 29, 437–447, doi:10.1016/j.jmpt.2006.06.003.
26. Fryer, G.; Morris, T.; Gibbons, P. Paraspinal Muscles and Intervertebral Dysfunction: Part One. J Manipulative Physiol Ther 2004, 27, 267–274, doi:10.1016/j.jmpt.2004.02.006.
27. Fryer, G. Somatic Dysfunction: An Osteopathic Conundrum. International Journal of Osteopathic Medicine 2016, 22, 52–63, doi:10.1016/j.ijosm.2016.02.002.
28. D'Alessandro, G.; Cerritelli, F.; Cortelli, P. Sensitization and Interoception as Key Neurological Concepts in Osteopathy and Other Manual Medicines. Front. Neurosci. 2016, 10, doi:10.3389/fnins.2016.00100.
29. Tozzi, P. A Unifying Neuro-Fasciagenic Model of Somatic Dysfunction – Underlying Mechanisms and Treatment – Part I. Journal of Bodywork and Movement Therapies 2015, 19, 310–326, doi:10.1016/j.jbmt.2015.01.001.
30. Verzella, M.; Affede, E.; Di Pietrantonio, L.; Cozzolino, V.; Cicchitti, L. Tissutal and Fluidic Aspects in Osteopathic Manual Therapy: A Narrative Review. Healthcare (Basel) 2022, 10, 1014, doi:10.3390/healthcare10061014.
31. Lunghi, C.; Tozzi, P.; Fusco, G. The Biomechanical Model in Manual Therapy: Is There an Ongoing Crisis or Just the Need to Revise the Underlying Concept and Application? Journal of Bodywork and Movement Therapies 2016, 20, 784–799, doi:10.1016/j.jbmt.2016.01.004.
32. Lunghi, C.; Baroni, F.; Amodio, A.; Consorti, G.; Tramontano, M.; Liem, T. Patient Active Approaches in Osteopathic Practice: A Scoping Review. Healthcare 2022, 10, 524, doi:10.3390/healthcare10030524.
33. Lunghi, C.; Consorti, G.; Tramontano, M.; Esteves, J.E.; Cerritelli, F. Perspectives on Tissue Adaptation Related to Allostatic Load: Scoping Review and Integrative Hypothesis with a Focus on Osteopathic Palpation. J Bodyw Mov Ther 2020, 24, 212–220, doi:10.1016/j.jbmt.2020.03.006.
34. Baroni, F.; Tramontano, M.; Barsotti, N.; Chiera, M.; Lanaro, D.; Lunghi, C. Osteopathic Structure/Function Models Renovation for a Person-Centered Approach: A Narrative Review and Integrative Hypothesis. J Complement Integr Med 2021, doi:10.1515/jcim-2021-0430.
35. Hohenschurz-Schmidt, D.; Thomson, O.P.; Rossettini, G.; Miciak, M.; Newell, D.; Roberts, L.; Vase, L.; Draper-Rodi, J. Avoiding Nocebo and Other Undesirable Effects in Chiropractic, Osteopathy and Physiotherapy: An Invitation to Reflect. Musculoskeletal Science and Practice 2022, 62, 102677, doi:10.1016/j.msksp.2022.102677.
36. Fryer, G. Intervertebral Dysfunction: A Discussion of the Manipulable Spinal Lesion. Journal of Osteopathic Medicine 2003, 6, 64–73, doi:10.1016/S1443-8461(03)80016-3.
37. Lucas, N.; Moran, R. What Is the Significance of Somatic Dysfunction in a Multicausal Model of Aetiology? International Journal of Osteopathic Medicine 2005, 8, 39–40, doi:10.1016/j.ijosm.2005.04.003.
38. Moran, R. Somatic Dysfunction – Conceptually Fascinating, but Does It Help Us Address Health Needs? International Journal of Osteopathic Medicine 2016, 22, 1–2, doi:10.1016/j.ijosm.2016.11.001.
39. Esteves, J.E.; Zegarra-Parodi, R.; Dun, P. van; Cerritelli, F.; Vaucher, P. Models and Theoretical Frameworks for Osteopathic Care – A Critical View and Call for Updates and Research. International Journal of Osteopathic Medicine 2020, 35, 1–4, doi:10.1016/j.ijosm.2020.01.003.
40. Basile, F.; Scionti, R.; Petracca, M. Diagnostic Reliability of Osteopathic Tests: A Systematic Review. International Journal of Osteopathic Medicine 2017, 25, 21–29, doi:10.1016/j.ijosm.2017.03.004.
41. Guillaud, A.; Darbois, N.; Monvoisin, R.; Pinsault, N. Reliability of Diagnosis and Clinical Efficacy of Visceral Osteopathy: A Systematic Review. BMC Complementary and Alternative Medicine 2018, 18, 65, doi:10.1186/s12906-018-2098-8.
42. Guillaud, A.; Darbois, N.; Monvoisin, R.; Pinsault, N. Reliability of Diagnosis and Clinical Efficacy of Cranial Osteopathy: A Systematic Review. PLOS ONE 2016, 11, e0167823, doi:10.1371/journal.pone.0167823.
43. Noy, M.; Macedo, L.; Carlesso, L. Biomedical Origins of the Term "osteopathic Lesion" and Its Impact on People in Pain. International Journal of Osteopathic Medicine 2020, 37, 40–43, doi:10.1016/j.ijosm.2020.06.002.

44. Lunghi, C.; Baroni, F. Cynefin Framework for Evidence-Informed Clinical Reasoning and Decision-Making. Journal of Osteopathic Medicine 2019, 119, 312–321, doi:10.7556/jaoa.2019.053.
45. Baroni, F.; Ruffini, N.; D'Alessandro, G.; Consorti, G.; Lunghi, C. The Role of Touch in Osteopathic Practice: A Narrative Review and Integrative Hypothesis. Complement Ther Clin Pract 2021, 42, 101277, doi:10.1016/j.ctcp.2020.101277.
46. Bergna, A.; Vismara, L.; Parravicini, G.; Dal Farra, F. A New Perspective for Somatic Dysfunction in Osteopathy: The Variability Model. J Bodyw Mov Ther 2020, 24, 181–189, doi:10.1016/j.jbmt.2020.03.008.
47. Tarantino, A.G.; Vismara, L.; Buffone, F.; Bianchi, G.; Bergna, A.; Vanoni, M.; Tabbi, C.; Bresesti, I.; Agosti, M. Model-Base Estimation of Non-Invasive Ventilation Weaning of Preterm Infants Exposed to Osteopathic Manipulative Treatment: A Propensity-Score-Matched Cohort Study. Healthcare 2022, 10, 2379, doi:10.3390/healthcare10122379.
48. International Classification of Functioning, Disability and Health (ICF) Available online: https://icd.who.int/dev11/l-icf/en (accessed on 24 November 2022).
49. Беляев, А.Ф. Остеопатия и Международная классификация функционирования, ограничений жизнедеятельности и здоровья (часть 1). Российский остеопатический журнал 2021, 0, 99–108, doi:10.32885/2220-0975-2021-1-99-108.
50. Беляев, А.Ф. Остеопатия и Международная классификация функционирования, ограничений жизнедеятельности и здоровья (часть 2). Российский остеопатический журнал 2021, 0, 86–97, doi:10.32885/2220-0975-2021-2-86-97.
51. Bettelli, L.; Pisa, V.; Formica, A. "I Do It My Way" - Italian Osteopaths' Beliefs and Attitudes about Five Osteopathic Models: A Qualitative Study. International Journal of Osteopathic Medicine 2020, 38, 57–64, doi:10.1016/j.ijosm.2020.10.009.
52. Tramontano, M.; Tamburella, F.; Dal Farra, F.; Bergna, A.; Lunghi, C.; Innocenti, M.; Cavera, F.; Savini, F.; Manzo, V.; D'Alessandro, G. International Overview of Somatic Dysfunction Assessment and Treatment in Osteopathic Research: A Scoping Review. Healthcare (Basel) 2021, 10, 28, doi:10.3390/healthcare10010028.
53. Ponzo, V.; Cinnera, A.M.; Mommo, F.; Caltagirone, C.; Koch, G.; Tramontano, M. Osteopathic Manipulative Therapy Potentiates Motor Cortical Plasticity. Journal of Osteopathic Medicine 2018, 118, 396–402, doi:10.7556/jaoa.2018.084.
54. Tamburella, F.; Piras, F.; Piras, F.; Spanò, B.; Tramontano, M.; Gili, T. Cerebral Perfusion Changes After Osteopathic Manipulative Treatment: A Randomized Manual Placebo-Controlled Trial. Front. Physiol. 2019, 10, doi:10.3389/fphys.2019.00403.
55. Cerritelli, F.; Chiacchiaretta, P.; Gambi, F.; Perrucci, M.G.; Barassi, G.; Visciano, C.; Bellomo, R.G.; Saggini, R.; Ferretti, A. Effect of Manual Approaches with Osteopathic Modality on Brain Correlates of Interoception: An FMRI Study. Sci Rep 2020, 10, 3214, doi:10.1038/s41598-020-60253-6.
56. Simmonds, N.; Miller, P.; Gemmell, H. A Theoretical Framework for the Role of Fascia in Manual Therapy. Journal of Bodywork and Movement Therapies 2012, 16, 83–93, doi:10.1016/j.jbmt.2010.08.001.
57. Fryer, G. Integrating Osteopathic Approaches Based on Biopsychosocial Therapeutic Mechanisms. Part 1: The Mechanisms. International Journal of Osteopathic Medicine 2017, 25, 30–41, doi:10.1016/j.ijosm.2017.05.002.
58. Fryer, G. Integrating Osteopathic Approaches Based on Biopsychosocial Therapeutic Mechanisms. Part 2: Clinical Approach. International Journal of Osteopathic Medicine 2017, 26, 36–43, doi:10.1016/j.ijosm.2017.05.001.
59. Lederman, E. A Process Approach in Osteopathy: Beyond the Structural Model. International Journal of Osteopathic Medicine 2017, 23, 22–35, doi:10.1016/j.ijosm.2016.03.004.
60. Dugailly, P.-M.; Fassin, S.; Maroye, L.; Evers, L.; Klein, P.; Feipel, V. Effect of a General Osteopathic Treatment on Body Satisfaction, Global Self Perception and Anxiety: A Randomized Trial in Asymptomatic Female Students. International Journal of Osteopathic Medicine 2014, 17, 94–101, doi:10.1016/j.ijosm.2013.08.001.
61. McGlone, F.; Cerritelli, F.; Walker, S.; Esteves, J. The Role of Gentle Touch in Perinatal Osteopathic Manual Therapy. Neuroscience & Biobehavioral Reviews 2017, 72, 1–9, doi:10.1016/j.neubiorev.2016.11.009.
62. Elkiss, M.L.; Jerome, J.A. Touch—More Than a Basic Science. Journal of Osteopathic Medicine 2012, 112, 514–517, doi:10.7556/jaoa.2012.112.8.514.
63. Consedine, S.; Standen, C.; Niven, E. Knowing Hands Converse with an Expressive Body – An Experience of Osteopathic Touch. International Journal of Osteopathic Medicine 2016, 19, 3–12, doi:10.1016/j.ijosm.2015.06.002.
64. Gyer, G.; Michael, J.; Inklebarger, J.; Tedla, J.S. Spinal Manipulation Therapy: Is It All about the Brain? A Current Review of the Neurophysiological Effects of Manipulation. Journal of Integrative Medicine 2019, 17, 328–337, doi:10.1016/j.joim.2019.05.004.
